# Genistein suppresses FLT4 and inhibits human colorectal cancer metastasis

**DOI:** 10.18632/oncotarget.3064

**Published:** 2014-12-18

**Authors:** Xiao Xiao, Zhiguo Liu, Rui Wang, Jiayin Wang, Song Zhang, Xiqiang Cai, Kaichun Wu, Raymond C. Bergan, Li Xu, Daiming Fan

**Affiliations:** ^1^ State Key Laboratory of Cancer Biology and Xijing Hospital of Digestive Diseases, Xijing Hospital, Fourth Military Medical University, Xi'an 710032, Shaanxi Province, PR China; ^2^ The Genome Institute, Washington University in St. Louis, St. Louis, MO, USA; ^3^ Department of Medicine, Robert H. Lurie Cancer Center and Center for Drug Discovery and Chemical Biology of Northwestern University, Chicago, IL, USA

**Keywords:** colorectal cancer, genistein, FLT4, metastasis, orthotopic mouse model

## Abstract

Dietary consumption of genistein, found in soy, has been associated with a potentially protective role in colorectal cancer (CRC) development and progression. Herein we demonstrate that genistein will inhibit human CRC cell invasion and migration, that it does so at non-cytotoxic concentrations and we demonstrate this in multiple human CRC cell lines. After orthotopic implantation of human CRC tumors into mice, oral genistein did not inhibit tumor growth, but did inhibit distant metastasis formation, and was non-toxic to mice. Using a qPCR array, we screened for genistein-induced changes in gene expression, followed by Western blot confirmation, demonstrating that genistein downregulated matrix metalloproteinase 2 and Fms-Related Tyrosine Kinase 4 (FLT4; vascular endothelial growth factor receptor 3). After demonstrating that genistein suppressed neo-angiogenesis in mouse tumors, we examined FLT4 expression in primary CRC and adjacent normal colonic tissue from 60 human subjects, demonstrating that increased FLT4 significantly correlates with increased stage and decreased survival. In summary, we demonstrate for the first time that genistein inhibits human CRC metastasis at dietary, non-toxic, doses. FLT4 is identified as a marker of metastatic disease, and as a response marker for small molecule therapeutics that inhibit CRC metastasis.

## INTRODUCTION

Colorectal cancer (CRC) is the third leading cause of cancer-related death in developed countries [1]. Increased incidence of CRC has also been observed in developing countries, likely due to associated changes in diet and environment. The five-year survival rate exceeds 90% in patients diagnosed with early stage CRC, while it is less than 20% for those with metastatic CRC [2]. The development of metastasis is therefore a major determinant of survival. There are currently no treatments that selectively inhibit processes that drive metastasis. Thus, the discovery and development of a safe and effective drug that is able to inhibit human CRC metastasis remains an important goal.

It is widely known that there is a wide variation in cancer rates from country to country. In particular, Asians, who have historically consumed a traditional diet high in soy, have a low incidence of clinical CRC [3]. However, Asians who immigrate to the United States and adopt a Western diet have an increased incidence of CRC [4]. These findings demonstrate that dietary and/or lifestyle factors influence the incidence of CRC, and are consistent with the notion that soy consumption may be in part responsible. Further, there is a large body of epidemiologic evidence to suggest that diets containing high amounts of soy are associated with an overall low rate of CRC mortality [5-7]. Genistein (4′, 5, 7-trihydroxyisoflavone), which is present in high amounts in soy products, has been specifically evaluated in many of these studies, and is thought to represent a key bioactive component. It is reported that among the adult Chinese and Japanese populations, the average daily dietary intakes of genistein is 39 and 47 mg, respectively, whereas for those consuming a traditional Western diet, average daily consumption is only 1-2 mg/d [8-10]. As the incidence rate of CRC is historically much lower in Japan and China compared to Western countries, one possible explanation for this, at least in part, is that Asians consume much more genistein.

Across a variety of experimental models, genistein has shown anticancer activity, typically in association with the suppression of cell proliferation and/or induction of apoptosis [11]. In CRC, previous studies have shown that genistein can decrease cell proliferation [12], and can induce G2/M phase cell cycle arrest and apoptosis [13]. Other studies implicate genistein's role in carcinogenesis through the epigenetic modulation of DNA, including DNA promoter methylation and histone modification, resulting in altered miRNA expression patterns [14]. However, genistein's effects are concentration dependent, the majority of these effects are observed in conjunction with high concentrations of genistein, i.e., mid-to-high micromolar concentrations, and the plethora of reported effects has raised concerns about specificity. At lower concentrations, overlapping with those achieved in the blood of humans after dietary consumption, genistein has been shown to inhibit cell motility and metastasis of human prostate cancer [15, 16]. However, the role of genistein in other cancers in this regard remains to be defined. We therefore conducted the current study designed to determine whether genistein affected human CRC metastasis. Further, given the complexity of metastasis and our current inability to successfully therapeutically target it [16], we sought to use genistein as a probe to analyze the associated underlying molecular mechanisms.

Metastasis is a complex, multistep process made up of a cascade of sequential steps involving changes in cellular invasion, migration, adhesion, movement of cancer cells through the circulatory system, and their re-implantation within a separate organ located at a distant site in the body, followed by colonization, tumor growth and the associated formation of new capillaries. In order to successfully metastasize, cancer cells must overcome what have been identified as three major barriers [17, 18]. The first relates to cellular attachment to the extracellular matrix, and the basement membrane in particular. Cells must re-program themselves in order to survive when not stably attached. The second requires an increased capacity to produce proteases that are able to induce local degradation of the extracellular matrix. And the third involves the ability of cancer cells to migrate through such a modified matrix. Increased cell migration, coupled to increased extracellular matrix degradation, constitute the major components of the composite process of cellular invasion. Increased cell invasion is an essential characteristic of the metastatic phenotype, is absolutely necessary for cells to successfully traverse the metastatic cascade, and strategies designed to selectively inhibit this process are actively being pursued, but remain elusive. The ability to inhibit initial cell invasion would in essence prevent the development of the series of events downstream from it that together lead to metastasis. Therefore, we began to investigate the effects of genistein on cell invasion and migration at cellular level, and then moved on to test CRC metastasis, using *in vivo* models.

In the current study, we demonstrate for the first time that genistein inhibits CRC cell invasive and migratory ability, and that it does so at concentrations that are not toxic to cancer cells *in vitro*. Using a clinically relevant orthotopic implantation murine model, we demonstrate that genistein inhibits human CRC cell metastasis. Based upon these positive findings, we went on to use genistein as a chemical probe to deepen our understanding of associated regulatory mechanisms. From an upfront screen, we went on to demonstrate that genistein suppresses expression of matrix metalloproteinase 2 (MMP2) and of Fms-Related Tyrosine Kinase 4 (FLT4), and that it does so in cells *in vitro* and in tumor tissue *in vivo*. Focusing upon this newly identified role for FLT4, we demonstrate that its overexpression in human CRC tissue is associated with increased stage and early death from the development of metastatic CRC. We hereby identify genistein as an inhibitor of human CRC metastasis and an inhibitor of FLT4 expression. Further, we identify FLT4 as a potential marker for the development of metastatic CRC.

## RESULTS

### Genistein's effects on CRC cell viability

**Figure 1:**
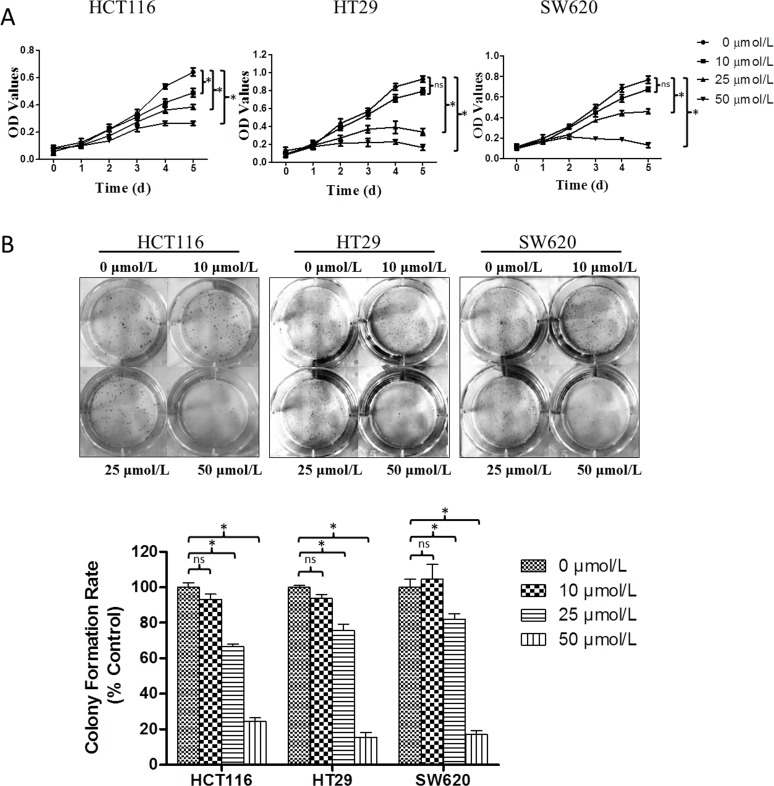
Genistein's effects on CRC cell viability (A) Effects of genistein on cell growth. HCT116, HT29 and SW620 cells were treated with 0, 10, 25 or 50 μmol/L genistein, and effects upon cell growth were measured daily over a five-day period. Data are the mean ± SEM OD value from a single experiment run in replicates of N=3; similar results were observed in a separate experiment, also N=3. * denotes p<0.05 comparing the cell viability at the fifth day to control group (P, by student's T TEST). (B) Effects of genistein on colony formation. One day after pretreatment at the indicated concentrations of genistein, cells were plated into 6-well plate at 1000 cells per well in the absence of genistein after 10 days, colonies counted. Data represented the mean ± SEM, from a single experiment run in replicates of N=3, and expressed as the percent of control (i.e., 0 μmol/L/vehicle only); similar results were observed in a separate experiment, also N=3. One-way ANOVA was used to calculate the significance of difference among groups. SNK analysis was used to compare differences between two groups as indicated in the figure. ns denotes p>0.05, * denotes p<0.05 compared to control group.

As induction of cell death can falsely affect measurement of cell movement, our initial investigations sought to characterize the concentration of genistein that was not toxic to cells. We first performed a cell proliferation assay on HCT116, HT29 and SW620 cells, treated with different concentrations of genistein, and measured effects upon cell growth each day, for five days. As shown in Fig. 1A, genistein inhibited cell growth in a concentration- and time-dependent manner. At 10 μmol/L, no significant effects were observed until after 72 hr, and then they were only minor. In contrast, at 25 and 50 μmol/L, genistein inhibited cell growth at 48hr and decreased it by up to 83% at 5 days. We further corroborated these effects by performing colony formation assays. After one day pre-treatment with different concentrations of genistein, cells were cultured for another 10 days, and colonies counted. As depicted in Fig. 1B, 10 μmol/L genistein did not decrease colony formation, while both 25 μmol/L and 50 μmol/L genistein significantly decreased colony formation in a concentration-dependent manner, and did so in all three cell lines tested.

### Genistein inhibits CRC cell invasion and migration

**Figure 2:**
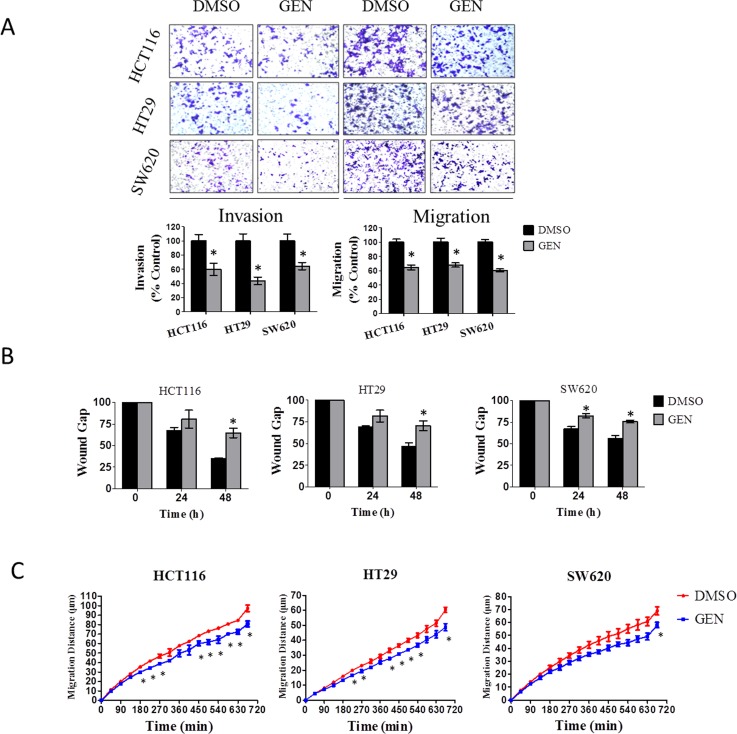
Genistein inhibits CRC cell invasion and migration (A) Transwell assay. Transwell assay was adopted to analyze cell invasion and migration of CRC cells. Cells on the lower side of the transwell membrane were counted. Representative images of different cell lines under different treatment conditions, stained with crystal violet and originally imaged at 200× magnification, are depicted. Data depicted graphically are the mean ± SEM of a single experiment, similar results were obtained in a separate experiment performed at a separate time, each experiment was in replicates of N=3. * denotes p<0.05 compared to vehicle control group by Student's t test. (B) Wound healing assay. Cells were pretreated with 10 μmol/L genistein or vehicle for one day, the wound created, and wound closure over 48 hr was measured. The wound gap was calculated to reflect cell migration degree. The area of 0 hr was set as 100 percent, and the percent changes at 24-hr and 48-hr time point were recorded. Data represented the mean ± SEM of a single experiment, each in replicates of N=3. Similar results were obtained in a separate experiment performed at a separate time, also in replicates of N=3. * denotes p<0.05 compared to control group by student's t test. (C) Cell migration assay. After pretreatment of cells with 10 μmol/L genistein for one day, average cell migration distance was recorded every 45 min over a 12-hr period of time as described in materials and methods. The data presented as one representative mean ± SEM from at least three separate experiments. * denotes P < 0.05 compared to control by student's t test. GEN: genistein.

For cell invasion and migration experiments, cells were treated with 10 μmol/L genistein for a total of 48hr (24hr pre-treatment plus 24hr during invasion or migration). As indicated above, this concentration has no impact upon cell viability at 48hr, and even with continued treatment at 10 μmol/L, no effects are observed for another 48 hr (i.e., at the 96 hr time point), and are even then only minor. Cell invasion was measured by a matrigel™ transwell invasion assay. As shown in Fig. 2A, genistein significantly inhibited cell invasion and cell migration in all three cell lines tested, inhibiting cell invasion by 36% to 56%, and inhibiting cell migration by 32% to 39%.

We further corroborated these findings in a wound healing assay and in the Cellomics high content cell migration system. The wound assay is shown in Fig. 2B, and demonstrates that genistein significantly inhibits wound closure. With the Cellomics high content system, we tracked and quantified the movement of individual cells across a two dimensional surface over a 12-hr period of time. As can be seen in Fig. 2C, genistein significantly inhibited the migration of all three cell lines tested, with statistically significant effects detected as early as 180 min.

### Genistein effects on distant organ metastasis in orthotopic nude mouse model

**Figure 3:**
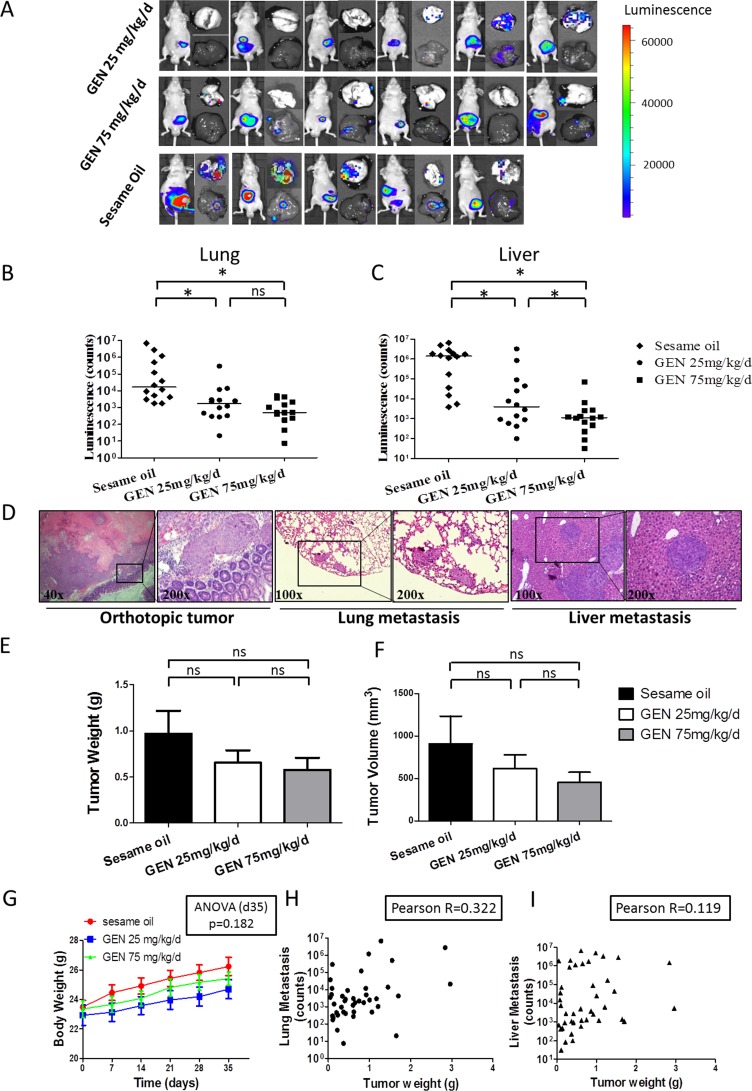
Genistein effects on distant organ metastasis in orthotopic nude mouse model Cohorts of 14 mice were orthotopically implanted with CRC cells. Images from luminescent imaging by IVIS system at the end of experiment were taken. The photon counts were automatically calculated by the software installed with the instrument. All exposure time and imaging parameters were set equally to generate comparable results. (A) Representative bioluminescent images from first batch of study with 6 nude mice in each cohort were presented. Left, whole mouse, upper right, lung, lower right, liver. The color scale depicts the photon flux (p/s) emitted. (One mouse in the control group died before the end of experiment.) (B, C) Imaging results of whole lung (B) and liver (C) organs were transformed to scatter plot scheme and the horizontal bar represented median value of each group. The Y axis was formalized as Log10 scale. Data from two batches studies, including total 14 mice in each group were presented. One-way ANOVA was used to calculate the significance of difference among groups. SNK analysis was used to compare differences between each two groups. ns denotes p>0.05. * denotes p<0.05 (D) Representative H&E staining of tissues from mice were displayed to show orthotopic tumor and metastatic loci in lung and liver. (E, F) Primary tumors formed by orthotopic implantation were separated and the weight (E) and volume (F) from each tumor were measured. Tumor volume was calculated as 0.52× (width) ^2^× (length). The mean ± SEM value from each cohort (sesame oil, 25 mg/kg/d genistein and 75 mg/kg/d) was presented as bar chart. One-way ANOVA was used to calculate the significance of difference among groups SNK analysis was used to calculate significance of differences between each two groups. ns denotes p>0.05. (G) Mice weight was recorded every week after surgery. The mean ± SEM value of each cohort was presented. (P>0.05 by one-way ANOVA). (H, I) Correlation between tumor weight and distant organ metastasis was showed. The graph depicts the metastatic image signal at lung (H) or liver (I) plotted against the tumor weight for each mouse. The Pearson R between these two parameters was determined. GEN: genistein.

The above findings demonstrate that genistein can inhibit the migration and invasion of CRC cells at concentrations below which cell toxicity is seen. Given the central role of cell migration and invasion in driving metastasis, we hypothesized that genistein would inhibit human CRC metastasis. We tested this in an orthotopic implantation model of human CRC cells in mice. This model closely recapitulates human disease in that it requires cells to complete all of the steps in the metastatic cascades, including initial steps, such as neo-angiogenesis formation, invasion out of the colon tract, as well as latter steps involving formation of distant metastasis. Specifically, in this experiment, small pieces of tissues from subcutaneous CRC tumors, arising from subcutaneously implanted cells, were transplanted into the cecal wall. Three days after orthotopic transplantation, 25 mg/kg/d or 75 mg/kg/d genistein or sesame oil (as control) was orally administered 5 days a week until the end of the experiment. After treatment for 5 weeks, primary tumor size and metastases were measured. Metastases were quantified by measuring bioluminescence in whole lung and liver organs immediately after necropsy (Fig. 3A).

As can be seen in Figs. 3B and C, genistein significantly inhibited metastasis to both lung and liver in a dose-dependent manner (ANOVA p < 0.001). With lung, metastases were significantly lower in 25 and 75 mg/kg/d groups, as compared to control (SNK p < 0.05, respectively). However, the difference between 25 and 75 mg/kg/d groups was not significant (SNK p = 0.226). With liver, metastases were also significantly lower in 25 and 75 mg/kg/d groups, as compared to control (SNK p < 0.05, respectively), and metastases in the 75 mg group were significantly lower than those of the 25 mg group (p < 0.05). We did not find ascites in any of our mice, although it was reported in other studies that ascites appeared after orthotopic tumor implantation [19, 20]. The existence of primary tumors and metastatic loci in lung and liver were confirmed by H&E staining (Fig. 3D). We also measured primary tumor weight and size for each mouse. As shown in Figs. 3E and F, genistein treated groups showed a tendency toward reduced tumor weight (ANOVA p value = 0.276) and tumor size (ANOVA p value = 0.354), but the difference was not significant. Together, these findings demonstrate that genistein inhibits human CRC metastasis in a dose-dependent manner.

Our previous study with genistein on mice using doses at and above current doses, comprehensively evaluated the systemic side effects of genistein in mice, failed to find any toxicity [21]. For the current study, we therefore only measured body weight, in addition to observing animal behavior. Genistein had no effect upon behavior, nor upon body weight (Fig. 3G). These findings indicate that genistein did not exert systemic toxicity in mice at the given doses.

It is recognized that the propensity to metastasize increases as tumor mass increases. As we found a tendency for reduced tumor weight by genistein, this raises the possibility that the differences in metastasis observed in the current study might just result from differences in tumor mass. If this were the case, then tumor mass should closely correlate with degree of metastasis. We went on to demonstrate that there was a poor correlation between tumor mass and metastasis at the individual mouse level (Fig 3H, I). A weak positive correlation trend was noticed between tumor weight and lung metastasis (Pearson R=0.322, 95% CI: 0.020 to 0.570 P=0.038, R^2^=0.104), whereas the result for liver was not significant (Pearson R=0.119, 95% CI: −0.192 to 0.408 P=0.455, R^2^=0.014). These findings indicate that the anti-metastatic effect of genistein results from factors other than primary tumor growth.

### Genistein's effects on angiogenesis and cell proliferation *in vivo*

**Figure 4:**
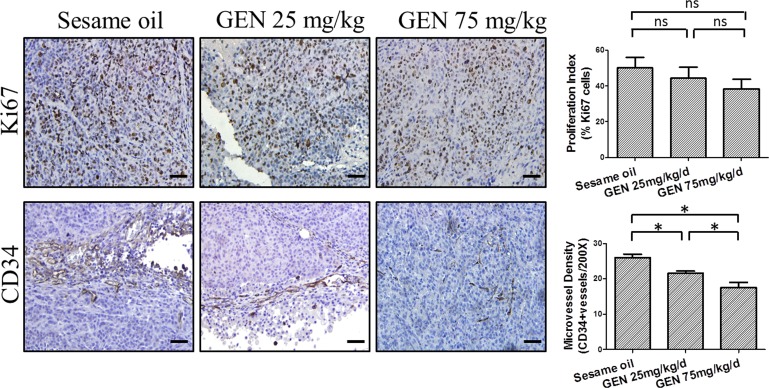
Genistein's effects on angiogenesis and cell proliferation *in vivo* Representative images of Ki67 and CD34 IHC staining from each cohort were displayed. The percentage of Ki67 in each specimen was calculated and the microvessel density was determined by the average number of positive CD34 staining in five random selected 200× fields. Data represent mean ± SEM for all mice in a given cohort. Scale bar: 50 μm. One-way ANOVA was used to calculate the significance of difference among groups. For CD34 staining, Games-Howell analysis was used to compare differences between two groups. For Ki67 staining, SNK analysis was used to compare differences between two groups. * denotes p<0.05 compared to controls. ns denotes p>0.05 compared to controls. GEN: genistein.

We next examined expression in orthotopic tumor tissues of the cell proliferation marker, Ki67, and the angiogenesis marker, CD34, by immunohistochemistry. Our results showed that although the percentage of Ki67 positive tumor cells showed a decreased trend after genistein treatments, the decreases were not statistically significant (one-way ANOVA p value =0.126) (Fig. 4, upper panel). This was in agreement with our above findings that no significant effect upon tumor weight was observed. Further, both of these findings are consistent with our *in vitro* results, which demonstrate the ability to achieve anti-motility effects in the absence of cell toxicity. However, genistein did significantly reduce microvessel density, as reflected by decreased CD34 staining in tumor tissues, and it did so in a dose-dependent manner, with the strongest reduction observed in the 75 mg/kg/d group (p=<0.000) (Fig. 4, lower panel).

### Identification of metastasis-related genes affected by genistein

**Figure 5:**
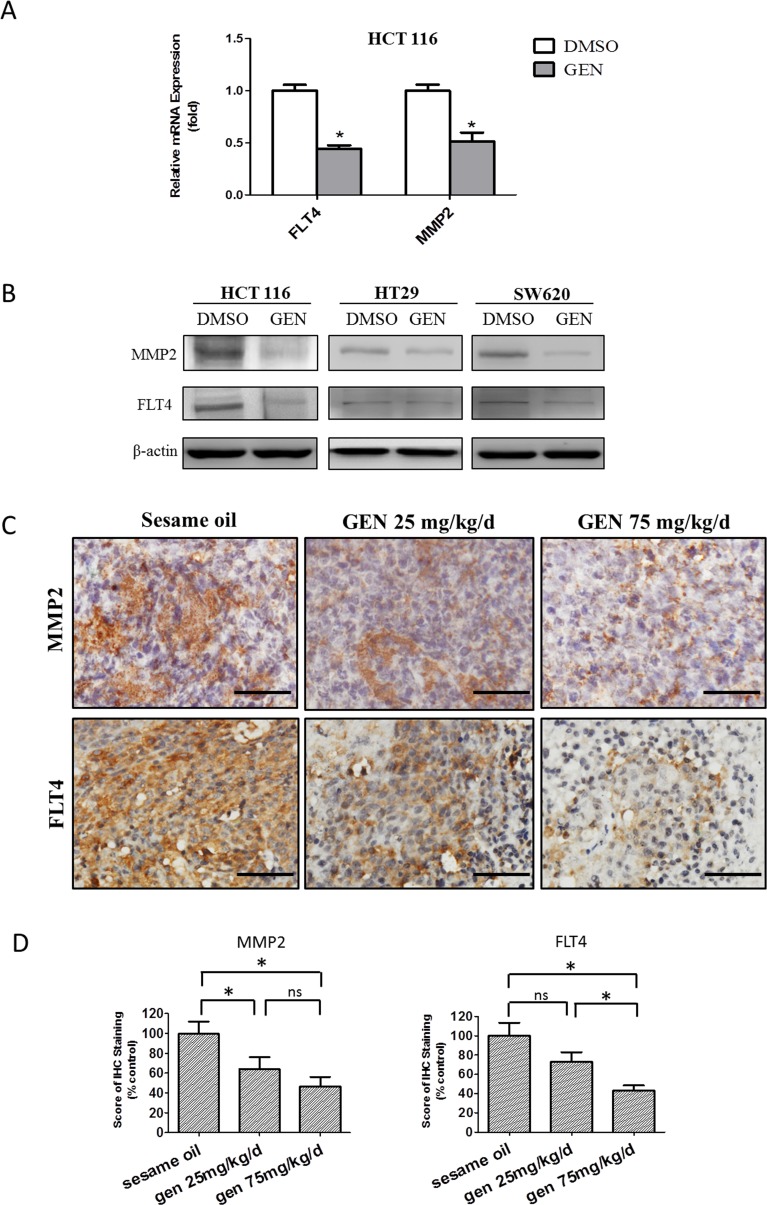
Identification of metastasis-related genes affected by genistein (A) The mRNA level of FLT4 and MMP2 was evaluated by qRT/PCR in HCT116 cells after genistein/DMSO treatment. Data represented the mean ± SEM of a single experiments, each in replicates of N=3. Similar results, normalized to 18S respectively, was assessed in at least 2 independent experiments, each in replicates of N=3. All control values were normalized to 1.0. * denotes p<0.05 compared to controls. (B) Protein level of FLT4 and MMP2 was measured by western blot in HCT116, HT29 and SW620 cells and β-actin served as the internal control. Depicted blots are representative, with similar results seen at least in one separate experiment. (C) MMP2 and FLT4 levels were studied in tumor tissue sections acquired from *in vivo* study by IHC staining and the representative images of IHC staining from each cohort were shown. Scale bar: 50 μm. (D) The expression level of MMP2 (left panel) and FLT4 (right panel) in tumor tissue sections was evaluated according to the scoring procedures described in materials and methods. Results were presented as a percentage ratio with the control group set as 100%. One-way ANOVA was used to calculate the significance of difference among groups, SNK analysis was used to calculate significance of differences between each two groups. * denotes p<0.05 compared with control group. ns denotes p>0.05 compared with controls. GEN: genistein.

Having demonstrated genistein's ability to inhibit human CRC metastasis, we next sought to use it as a chemical probe to better understand the molecular regulation of CRC metastasis. We approached this by treating HCT116 cells with/without 50 μmol/L genistein for 1day, followed by screening for altered gene expression on an 84-gene human tumor metastasis PCR array platform. Genes with more than twofold changes were considered of interest. Based upon this, 8 out of the 84 genes were downregulated, while 4 were upregulated (Table S1). We then tested these 14 genes by qPCR and confirmed MMP2 and FLT4 RNA levels were decreased by genistein (Fig. 5A). Next, we examined the effect of genistein on MMP2 and FLT4 protein expression in HCT116, HT29, and SW620 cells by Western blot (Fig. 5B). In all three cell lines tested, genistein decreased the expression of both MMP2 and FLT4 proteins. To check if this was the case *in vivo*, we measured protein expression in tissue sections from tumors of genistein treated or control mice by immunohistochemistry. As seen from Figs. 5C and D, genistein significantly decreased both MMP2 and FLT4 expression in a dose-dependent manner. Taken together, these findings demonstrate that genistein selectively suppresses MMP2 and FLT4 expression, and that it does so both *in vitro* and *in vivo*.

### Prognostic role of FLT4 in human CRC

**Figure 6:**
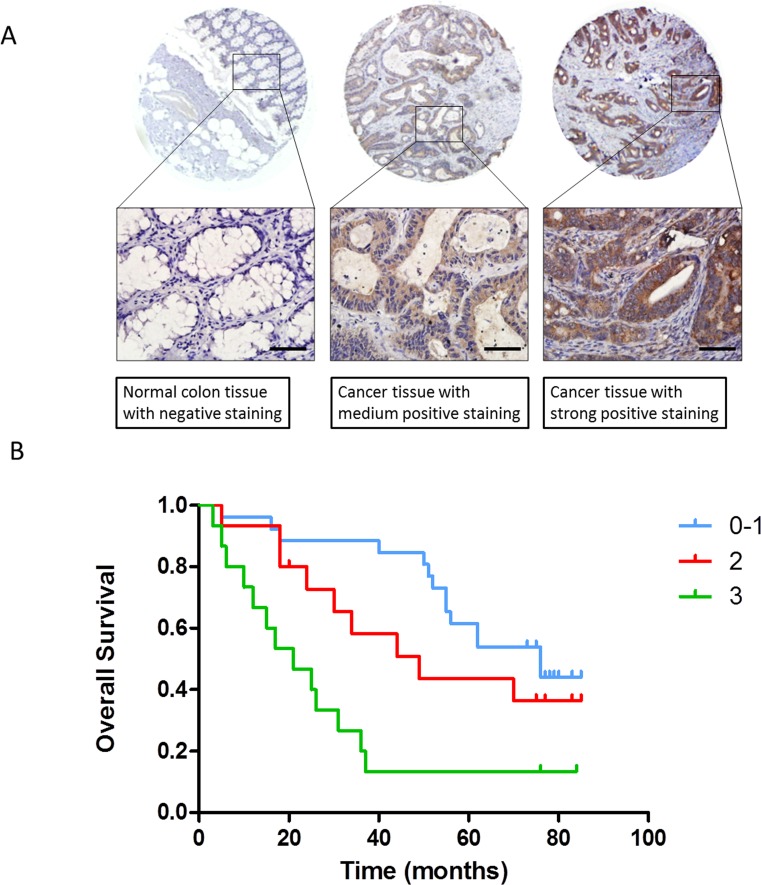
Prognostic role of FLT4 in human CRC (A) Representative images of normal colon tissue with negative staining (left), cancer tissue with medium (middle) or strong staining (right) were displayed. Scale bar: 50 μm. (B) Kaplan-Meyer method was used to analyze relationship between FLT4 expression and patients' prognosis. Increased expression of FLT4 indicated worse overall survival (P=0.001).

The above findings demonstrate that genistein decreases MMP-2 and FLT4 expression coincident with inhibition of CRC metastasis. MMP-2 has a well-established role in regulating cancer cell invasion and metastasis, in a variety of cancer types (32, 33), including CRC. Thus, while of clear importance to cancer, and to invasion and metastasis in particular, its role in this regard is relatively ubiquitous. Further, its function as an extracellular proteinase has been particularly difficult to therapeutically target with any type of selective efficacy. In contrast, FLT4 is an intracellular kinase, and little is known about its role in regulating metastasis, and CRC metastasis in particular. We therefore focused further investigations on FLT4. We utilized a colon human tissue array to examine the relationship between FLT4 expression and clinicopathological characteristics. This array included 60 cases of colon cancer tissues and normal colon mucosal tissues from the same patient diagnosed with colon adenocarcinoma. In this manner, we found that FLT4 expression level was significantly increased in CRC compared to that in paired non-cancerous tissues (Table S2). Furthermore, increased FLT4 was found to correlate with advanced clinical stage and with the presence of lymph node metastasis, but not with gender and age (Table 1, Fig. 6A). We then went on to analyze the relationship between FLT4 expression and patient prognosis by the Kaplan-Meier method (Fig. 6B). Patients with higher FLT4 expression had a statistically significant worse prognosis. Specifically, among 60 patients examined, those exhibiting weak, moderate and strong staining had a median survival of 76, 49 and 21 months respectively (p value =0.001). These results demonstrate that increased FLT4 is a poor prognostic marker for CRC, and implicate it in CRC progression.

**Table 1:** Clinic pathological association of FLT4 in colon cancer

Characteristic	Number of specimens	FLT4 expression				P value
		0	1	2	3	
Gender						0.498
Male	31	3	12	7	9	
Female	29	6	7	8	8	
Age						0.774
≤70	20					
>70	40					
TNM Stage						0.031
I	5	2	3	0	0	
II	31	5	13	6	7	
III	22	2	3	9	8	
IV	2	0	0	0	2	
Lymph node metastases						0.013
0	36	7	16	6	7	
≥1	24	2	3	9	10	

χ^2^ test was used to analyze the relationship of FLT4 expression and clinicopathological factor. P values less than 0.05 were considered significant.

## DISCUSSION

We demonstrate for the first time that genistein can inhibit the invasion, migration and metastasis of CRC cells. Further, we accomplished this with human CRC cells. Importantly, we demonstrate this function *in vitro* at concentrations that are non-toxic to cells. The non-toxic nature of therapy in association with therapeutic efficacy is further supported by findings in our systemic murine models. Specifically, we observed anti-metastatic efficacy in a dose-responsive fashion, while observing no evidence of systemic toxicity. Further, genistein did not significantly inhibit tumor growth, nor did it inhibit cell growth, as assessed by Ki67 expression. These *in vivo* findings directly support our *in vitro* ones, which demonstrate that anti-motility effects can be induced at concentrations that do not induce toxic effects. Finally, we further corroborated the specificity of *in vivo* findings by going on to demonstrate that there was a poor correlation between tumor size and number of metastasis.

It is also of importance to note that anti-motility efficacy is observed *in vitro* at concentrations that approximate those attainable in the blood with administration of dietary doses to humans. Further, we demonstrate anti-metastatic efficacy in a murine model in which genistein is delivered via the oral route, as it would be through dietary consumption. Finally, we know from prior work by us that the doses we administered to mice provide blood concentrations that directly overlap with those attained by dietary consumption [22-24]. The clinical relevance of our findings is further supported by the fact that efficacy was observed at genistein dosages taken daily with Eastern-style diet or Western-style diet supplemented with genistein [10, 24]. As our further analysis of primary tumor weight with the occurrence of lung or liver metastasis did not reveal their close correlation, our findings indicate that inhibition of CRC metastasis by genistein is not dependent on primary tumor growth. Together, these findings provide a mechanistic explanation for the lower incidence of clinical, i.e., metastatic, CRC observed in high soy consuming populations. Based upon these findings, it will be important to begin evaluating this potential mechanism in humans, and to compare findings in cohorts who consume high soy versus those who do not.

Also, having demonstrated genistein's therapeutic efficacy, this led us to use genistein as a chemical probe. These studies were successful in that we found that genistein decreased expression of MMP-2 and FLT4. The finding that genistein decreased MMP-2 expression served as an important positive control for these studies. This is because MMP-2 has been widely implicated in cancer cell invasion and metastasis in a wide array of cancer types, including CRC (32, 33). Further, genistein has been shown to decrease MMP-2 expression in human prostate cancer, coincident with its ability to inhibit human prostate cancer cell invasion [25, 26].

Our identification that genistein decreased FLT4 expression was considered of particularly high potential importance by us. FLT4 is also known as vascular endothelial growth factor receptor 3 (VEGFR3), and it has been implicated in cancer related to its role in increasing neo-angiogenesis [27]. Previous work on the receptor has produced variable findings, with some studies identifying its expression in tumors but failing to find any correlation between level of expression and clinicopathological parameters [28], whilst several studies do report a positive and significant correlation between the level of FLT4 expression in the tumor and the development of metastasis and poor prognosis [29, 30]. Our results point to a significant positive correlation between increased FLT4 expression and an aggressive tumor phenotype and resultant poor survival. Specifically, we demonstrated that elevated FLT4 was associated with lymph-node metastasis, advanced stage, and with early death from CRC. Our findings are in agreement with those drawn from the studies of others involving a larger sample analysis [29, 30]. Furthermore, our findings are in agreement with the current biological role of the VEGF family of receptors, inclusive of FLT4, as drivers of neo-angiogenesis, and resultant metastasis. Our identification of FLT4 as an important regulator of cancer metastasis is supported by the work of others who report that the VEGF-C/FLT-4 axis promotes lung cancer cell migration and invasion [31]. Taken together, our findings implicate FLT4 in the regulation of CRC progression to a metastatic phenotype. It will be important for future studies to investigate the specific mechanism by which FLT4 acts to stimulate metastasis. Considering that FLT4 is a protein kinase, it raises the notion that a FLT4 specific kinase inhibitor may have significant anti-metastatic potential in CRC. In this regard, we want to highlight that although genistein is a known protein kinase inhibitor, it in fact decreased FLT4 expression at the transcript level, resulting in decreased protein expression. Therefore, it is unlikely that genistein-mediated inhibition of FLT4 kinase activity is relevant.

Of interest, genistein decreased tumor-associated angiogenesis. There are several potential mechanisms that may be responsible for this finding. A likely one involves a direct extension of genistein's anti-motility action. In particular, it may be inhibiting endothelial cell movement. This notion is further supported by the fact that genistein decreases FLT4 expression. Other potential mechanisms include modulation of microenvironmental cytokines, altering epithelial to mesenchymal cell transition, as well as others. While our findings were the first to demonstrate that genistein inhibited angiogenesis in CRC, it has previously been shown to do so in other cancer types, including bladder and hepatocellular carcinoma [32, 33]. Importantly, our identification of genistein-mediated suppression of FLT4 serves to provide a mechanistic explanation for its antiangiogenic action across several different tumor types. The process of neo-angiogenesis has been recognized as a vital factor for sustaining tumor growth [34]. Our finding that genistein effectively suppressed formation of neo-angiogenesis has significant implications for the therapeutic use of genistein in humans. Anti-angiogenesis drugs, such as bevacizumab (anti-VEGF monoclonal antibody), have already been successfully approved for clinically treating many malignant tumors, including CRC. Nevertheless, the side effects associated with bevacizumab is a real concern clinically [35, 36]. Thus, the identification of novel antiangiogenic agents with less side effects, such as genistein, is urgently needed, and should be further pursued in this regard in future studies.

Altogether, in this study, we demonstrate for the first time that genistein is able to selectively inhibit human CRC cell motility and metastasis. We also demonstrate that genistein exerts such inhibitory effects at concentrations that approximate those attained with dietary intake. As such, our findings provide a solid mechanistic rational for epidemiologic studies which associate soy consumption with decreased metastasis. We demonstrate that genistein suppresses MMP2 and FLT4, coincident with inhibiting cell motility and metastasis. Suppression of FLT4 is accompanied by inhibition of primary tumor neo-angiogenesis. Our findings provide a strong rationale for pursuing the clinical application of genistein to inhibit CRC metastasis. Moreover, based on our results, MMP2, FLT4 and CD34 could be used as biomarkers to monitor genistein efficacy in clinic.

## METHODS

### Cells

HCT116, SW620 and HT 29 colon cancer cell lines were purchased from The Cell Bank of Type Culture Collection of Chinese Academy of Sciences (Shanghai, China). All cells were cultured in DMEM supplemented with 10% fetal bovine serum (FBS). HCT 116-LUC cells (stable luciferase expression) were used for orthotopic transplantation, and were established by lentivirus infection, followed by puromycin selection.

### Cell proliferation assay

The CCK8 assay kit (Beyotime Institute of Biotechnology, Shanghai, China) was used to determine cell viability after genistein treatment. Briefly, cells were seeded in 96-well plate (1000 cells/well) overnight before complete medium containing different concentrations (0, 10, 25, and 50 μmol/L) of genistein was added. Then the cells were cultured for up to 5 days. At the end of each day, cell viability was measured by adding CCK8 agent at a 10 μL/100μL medium ratio. Cells were incubated for 2 hr before recording absorbance at 450nm using a Varioskan™ Flash Multimode Reader (Thermo Fisher Scientific, MA, US).

### Colony formation assay

The procedures were performed as previously described by us [37]. Briefly, cells were pretreated with genistein or DMSO at the indicated dose for 24 hr before seeding on 6-well plate at a density of 1000 cells per well. After 10 days culture, the colonies were visualized by 0.3% crystal violet staining for 15 minutes. Excess crystal violet was removed by rinsing the plate with PBS. The visible colonies were counted and the colony formation rate of each group was calculated with the control group set as 100%.

### Transwell assays

Transwell cell invasion and cell migration assays were used to evaluate both cell migration and invasion, as previously described by us [38]. Briefly, for invasion assays, 50,000 cells were pretreated with 10 μmol/L genistein or DMSO for one day before seeding in media without FBS into the upper chamber of each transwell, which was precoated with Matrigel. Media with 20% FBS was placed in the lower chamber and served as chemoattractant. Cells were allowed to invade for 24 hr with/without genistein treatment. After that, noninvasive cells on the upper surface of the membrane were removed by a cotton swab and cells on the lower surface of the membrane were fixed and stained with crystal violet. Invasive cells attached to the lower surface of the filter were visualized and photographed at 200× magnification using an Olymus BX51 microscope. Photographs of 3 random fields from 3 replicate wells were recorded, and the number of cells was counted. For migration assay, all procedures were the same as in invasion assays except that 25,000 cells were seeded in each chamber and there was no Matrigel coated on the membrane of the transwell chamber.

### Wound healing assay

Cells were pretreated with 10 μmol/L genistein or DMSO one day before assay. Then, the confluent monolayer of cells was wounded gently by scratching with a 200-μL tip along the diameter of the well followed by PBS rinsing to remove debris. Fresh media, containing either genistein or DMSO, were then added. For each well, at least 3 pictures were taken with a microscope at a magnification of 100× at 3 time points (0, 24, and 48 hrs) after scratching. The degree of wound healing was represented by the percentage of the non-covered wound area.

### Single cell migration assay

As described previously with modification [39], cells in log phase were seeded in 96 well plates (5000 cells/well) and incubated at 37°C to allow adhesion. After treating with 10 μmol/L genistein or DMSO for one day, cells were rinsed with serum free medium twice and stained with Hoechst 33342 for 15min at room temperature (RT). After rinsing, cells were imaged every 45 min over a 12-hr-period using Cellomics ArrayScan® VTI HCS Reader (Thermo Fisher Scientific), with an additional incubator to maintain and image cells at 37°C. Specifically, the instrument randomly selected 49 evenly distributed fields in each well and automatically calculated the average migration distance of each cell. Every group contained 5 separate wells, i.e., N=5 replicates.

### Orthotopic murine model of colorectal cancer metastasis

All the procedures involving animals were reviewed and protocols were approved by Xijing Hospital Animal Care and Use Committee. Six-to-eight week old, athymic, Balb/c mice were obtained from Vital River Laboratories (Beijing, China). To obtain subcutaneous tumor used for tissue transplantation, 3×10^6^ HCT116-LUC cells were subcutaneously injected on each flank and allowed to form tumors. Two weeks after injection, subcutaneous tumors were isolated, cut into 2 mm pieces and kept briefly on ice until orthotopic implantation. For orthotopic transplantation, mice were anesthetized with pentobarbital before sterilizing. A 2-cm left abdominal flank incision was made and the cecum was isolated and fixed. A partial thickness cut in the cecal wall was made by fine needle, and a tumor piece was sutured into the incision. After re-inserting the cecum, the abdomen was closed with single layer suture [40]. Mice were randomly separated into three groups including, vehicle control (sesame oil), high-dose genistein (75 mg/kg/d) and low dose genistein (25 mg/kg/d). Three days after surgery, mice were weighted, and therapy begun and was administered daily, 5 days per week (Monday through Friday), for 5 weeks. Throughout the experiment, mice were weighed weekly. After 5 weeks treatment, mice were injected intraperitoneally with D-Luciferin (Caliper Life Sciences, Hopkinton, MA, US), allowed to move about freely for 3 minutes to promote absorption of substrate, were then anesthetized by isoflourane and underwent whole body imaging using the IVIS imaging system (Caliper Life Sciences, Hopkinton, MA, US). As the strong signals from the orthotopic tumor masked the much weaker metastatic signals, mice were then immediately necropsied, lung and liver harvested as whole organs, and their bioluminescence signals separately captured by IVIS imaging. Organs, and tumor tissues, were fixed in formalin, paraffin embedded, sectioned and stained with hematoxylin-and-eosin (H&E), and used for immunohistochemical analysis, as indicated. The weight of orthotopic tumor was measured, and its volume was calculated as 0.52× (width) ^2^× (length) with measures taken in two perpendicular dimensions, as previously described [21].

### Immunohistochemical staining

All procedures were performed as previously described by us [21, 38, 41]. Briefly, formalin-fixed paraffin-embedded tissues were prepared in 4-μm sections. After performing dewaxing, rehydration, blocking endogenous peroxidase activity and antigen retrieval steps, sections were blocked with 10% normal goat serum at RT for 15 min and incubated with primary antibodies against MMP2, FLT4, Ki67 and CD34 (Abcam, Cambridge, MA, US) at 4°C overnight. Corresponding secondary antibodies, conjugated to HorseRadish Peroxidase (DAKO, Carpinteria, CA), were incubated at RT for 30 min. Staining achieved with a DAB kit (Zhongshan Golden Bridge Biotechnology Company, Beijing, China), per manufacturer's instructions. Microvessel density was determined on CD34 stained slides by counting five representative fields in each specimen under 200X magnification, as described [42]. Ki67 expression was determined as the percentage of positive cells in the representative areas examined in each specimen, as described by us [21]. The expression level of MMP2 and FLT4 was calculated by considering the ratio and intensity of staining [31]. The ratio score was determined as follows: 1 for <25%, 2 for 26-50%, 3 for 51-75%, 4 for >75%. And the intensity score was determined as follows: 1 for weak staining, 2 for moderate staining and 3 for a high level of staining. A final composite score ranging from 0-12 was calculated by multiplying the ratio score to the intensity score. The tissue array of human colon cancer and normal colon tissue was purchased from Xi'an Alena Biotechnology Company. Tissue arrays were constructed from tissue that had already been collected from patients undergoing standard-of-care treatment. Further, all tissue was de-identified, and no links back to the patient are available, nor will be attempted. Also, clinical data associated with each patient was provided by Alena Biotechnology. Here too, no patient identifier information is provided, all data has been de-identified, and no links back to patients are available, nor attempted. For FLT4 staining in the tissue array, the final result was recorded as negative staining=0 (score 0~2), weak staining=1 (score 3~5), moderate staining=2 (score 6~8) and strong staining=3 (score 9~12). All tissue was scored by a single person, and in a blinded fashion.

### PCR array

Human Tumor Metastasis PCR Array (APHS-028A; Super Array Inc.) provided by Kangcheng Gene Chip Company (Shanghai, China) was used. Total RNA was extracted from genistein treated HCT116 cells or DMSO treated control cells using TRIZOL® reagent (Invitrogen, Carlsbad, CA, US), followed by synthesis of cDNA using SuperScript. III Reverse Transcriptase, 10mM dNTPs Mix, oligo (dT) _18_ (Invitrogen) and RNase Inhibitor (Epicentre, Madison, WI, US), all per manufacturer's protocol. The mixture of cDNA template and a 2×Super Array PCR master mix was added to the wells of the PCR Array plate (384-well) containing the gene-specific primer sets before real-time PCR was performed. The PCR cycling conditions were as follows: 40 cycles of 95°C for 15 s, 60°C for 1min, and 72°C for 30s. Five housekeeping genes (ACTB/NM_001101, B2M/NM_004048, GAPDH/NM_002046, HPRT1/NM_000194 and RPLP0/NM_001002) were used as internal controls. The ΔCt value of each metastasis-related gene in each group was calculated. The differential expression of each gene was measured according to the comparative Ct method (ΔΔCt) [43], and the fold-change in difference between the genistein treated and DMSO treated control groups were compared. A fold-change greater than 2 was regarded as up-regulation, and a fold-change less than 0.5 as down-regulation.

### RNA extraction and qRT-PCR

Total RNA from the colon cancer cells was extracted using TRIZOL® reagent (Invitrogen) according to manufacturer's instructions, and 500 ng RNA of each sample was subjected to cDNA synthesis using TaKaRa PrimeScript RT reagent kit (TaKaRa Biotechnology, Dalian, China). A Roche Light Cycler 480 PCR machine and SYBR®Premix Ex Taq™ Green I (TaKaRa) were used for the real-time PCR. The PCR program consisted of 40 cycles of the following steps: 95°C for 5s and 60°C for 30s. The 18S mRNA was set as the internal control and the final expression level of each gene was normalized to the control group. Primer sequences used in real-time PCR were listed as follows: FLT4 forward: GCCATGTACAAGTGTGTGGTCTC, FLT4 reverse: ACTTGTAGCTGTCGGCTTGG; MMP2 forward: CTCATCGCAGATGCCTGGAA, MMP2 reverse: TTCAGGTAATAGGCACCCTTGAAGA; 18S forward: CGGCTACCACATCCAAGGAA, 18S reverse: GCTGGAATTACCGCGGCT.

### Western blotting

Protein isolation was performed as described by us [25]. Briefly, protein samples were prepared using RIPA lysis buffer (25 mmol/L Tris–HCl, pH7.5, 150 mmol/L NaCl, 1 mmol/L EDTA, 1% TritonX-100) containing protease inhibitor cocktail tablet (Roche Applied Science, Mannheim Germany). Proteins were separated by 12% Sodium dodecyl sulfate–poly-acrylamide gel electrophoresis and were transferred to a nitrocellulose membrane. After blocking with Tris-buffered saline containing 5% non-fat milk powder and 0.1% Tween-20 for 1 hr at RT, the membrane was incubated with anti-FLT4 (Cell Signaling Technology, Danvers, MA, US), anti-MMP2 ((Santa Cruz Biotechnology, Delaware, CA, USA) or anti-actin (Sigma-Aldrich, St. Louis, MO, US) at 4°C overnight. Goat anti-mouse secondary antibody (Boster, Wuhan, Hubei, China) was used to incubate the membrane for 1h at RT and enhanced chemiluminescence was then used to visualize protein bands in BIO-RAD ChemiDoc XRS Imaging system.

### Statistical analysis

Results were presented as the mean ± SEM and analyzed by two-sided Student's t test or one-way ANOVA for continuous variables, as indicated. The bioluminescence values reflecting liver and lung metastasis were transformed to logarithm with base 10 before subjecting to statistical analysis. Student-Newman-Keuls (SNK) or Games-Howell approach was used to perform two-group comparison after one-way ANOVA depending on the homogeneity of variances. To evaluate the association between tumor weight and metastatic burden, the Spearman correlation coefficient was used. The relation between FLT4 expression and clinicopathological parameters was analyzed by Pearson Chi-square test. The overall survival was calculated using Kaplan-Meier method. SPSS 19.0 software (SPSS Inc., Chicago, IL, US) was used for statistical analysis. Statistical significance was considered present for P-values less than 0.05.

## SUPPLEMENTARY MATERIAL AND TABLES


