# Supplementary material for: Genistein suppresses FLT4 and inhibits human colorectal cancer metastasis
Source: Oncotarget. 2014 Dec 18;6(5):3225–39. doi: 10.18632/oncotarget.3064 (PMC4413649; doi:10.18632/oncotarget.3064)
Supplement: Supplementary file 1 [file oncotarget-06-3225-s001.pdf]

Genistein suppresses FLT4 and inhibits human colorectal cancer metastasis

Supplementary Material

Table S1 mRNA expression of metastasis-related genes after genistein treatment

Genes	Description	Fold change
CDH6	Cadherin 6, type 2, K-cadherin (fetal kidney)	3.38
CTSK	Cathepsin K	0.38
FGFR4	Fibroblast growth factor receptor 4	0.47
FLT4	Fms-related tyrosine kinase 4	0.1
KISS1	KiSS-1 metastasis-suppressor	0.48
KISS1R	KISS1 receptor	0.5
MGAT5	Mannosyl (alpha-1,6-)-glycoprotein beta-1,6-N-acetyl-glucosaminyltransferase	2.69
MMP10	Matrix metallopeptidase 10 (stromelysin 2)	12.25
MMP2	Matrix metallopeptidase 2 (gelatinase A, 72kDa gelatinase, 72kDa type IV collagenase)	0.17
MMP7	Matrix metallopeptidase 7 (matrilysin, uterine)	0.16
NR4A3	Nuclear receptor subfamily 4, group A, member 3	2.17
TSHR	Thyroid stimulating hormone receptor	0.3

Table S2 Expression of FLT4 in colon cancer and paired noncancerous tissues

**Tissue type**	**Number of specimens**	**FLT4 expression**				**P value**
		**0**	**1**	**2**	**3**	
Normal tissue	60	21	24	10	5	0.005
Tumor tissue	60	9	19	15	17	

χ^2 test was used to evaluate the significance of difference between two groups. P value less than 0.05 was considered significant.
